# Oncolytic vaccinia virus GLV-1h68 exhibits profound antitumoral activities in cell lines originating from neuroendocrine neoplasms

**DOI:** 10.1186/s12885-020-07121-8

**Published:** 2020-07-06

**Authors:** Linus D. Kloker, Susanne Berchtold, Irina Smirnow, Julia Beil, Andreas Krieg, Bence Sipos, Ulrich M. Lauer

**Affiliations:** 1 Department of Internal Medicine VIII, Department of Medical Oncology and Pneumology, University Hospital Tuebingen, University of Tuebingen, Otfried-Mueller-Strasse 10, 72076 Tuebingen, Baden-Wuerttemberg Germany; 2 German Cancer Consortium (DKTK), German Cancer Research Center (DKFZ), 72076 Tuebingen, Germany; 3 Department of Surgery (A), Heinrich-Heine-University and University Hospital Duesseldorf, 40225 Duesseldorf, Germany

**Keywords:** Endocrine cancers, Virotherapy, Immunotherapy, Vaccinia virus, Neuroendocrine tumors

## Abstract

**Background:**

Oncolytic virotherapy is an upcoming treatment option for many tumor entities. But so far, a first oncolytic virus only was approved for advanced stages of malignant melanomas. Neuroendocrine tumors (NETs) constitute a heterogenous group of tumors arising from the neuroendocrine system at diverse anatomic sites. Due to often slow growth rates and (in most cases) endocrine non-functionality, NETs are often detected only in a progressed metastatic situation, where therapy options are still severely limited. So far, immunotherapies and especially immunovirotherapies are not established as novel treatment modalities for NETs.

**Methods:**

In this immunovirotherapy study, pancreatic NET (BON-1, QGP-1), lung NET (H727, UMC-11), as well as neuroendocrine carcinoma (NEC) cell lines (HROC-57, NEC-DUE1) were employed. The well characterized genetically engineered vaccinia virus GLV-1 h68, which has already been investigated in various clinical trials, was chosen as virotherapeutical treatment modality.

**Results:**

Profound oncolytic efficiencies were found for NET/NEC tumor cells. Besides, NET/NEC tumor cell bound expression of GLV-1 h68-encoded marker genes was observed also. Furthermore, a highly efficient production of viral progenies was detected by sequential virus quantifications. Moreover, the mTOR inhibitor everolimus, licensed for treatment of metastatic NETs, was not found to interfere with GLV-1 h68 replication, making a combinatorial treatment of both feasible.

**Conclusions:**

In summary, the oncolytic vaccinia virus GLV-1 h68 was found to exhibit promising antitumoral activities, replication capacities and a potential for future combinatorial approaches in cell lines originating from neuroendocrine neoplasms. Based on these preliminary findings, virotherapeutic effects now have to be further evaluated in animal models for treatment of Neuroendocrine neoplasms (NENs).

## Background

Neuroendocrine neoplasms (NENs) are rare tumors which are developing in widespread anatomical origins such as the pancreas, lung and intestine. Only the minority of tumors show hormonal functionality, so that approximately 70% of NENs are non-functional and therefore asymptomatic in early stages. Accordingly, patients frequently present only in late metastatic disease stages. This as well as the rising incidence makes NENs an upcoming challenge in oncology [1].

NENs are subclassified into neuroendocrine tumors (NETs) and poorly differentiated neuroendocrine carcinomas (NECs). Generally, surgery is the treatment of choice for NENs in an early, still localized stage. In addition to classical chemotherapy and radiation, somatostatin analogues, peptide receptor radiotherapy, small molecule compounds such as sunitinib or everolimus are available for unresectable NETs [2]. Treatment options for NECs are still often restricted to chemotherapy and radiation [3]. Further therapy options for unresectable tumors such as several multi-kinase inhibitors or peptide receptor chemoradionuclide therapy are under development [4, 5] and also new therapeutic targets and treatment combination strategies are under extensive preclinical investigation [6].

Only very few approaches using oncolytic virotherapy in NEN treatment have been described so far [7–10]: oncolytic viruses (OV) are engineered to specifically target tumor cells, to produce enormous amounts of viral progeny within and thus to damage them harshly, resulting in significant rates of tumor cell lysis, i.e. oncolysis. Furthermore, infections by OV were found to turn immunosuppressive “cold” tumor microenvironments into “hot” ones by attracting a significant influx of immune cells. As a result, profound and long-lasting antitumoral immune responses can be induced.

The oncolytic virus employed in this study is a genetically modified DNA virus which has already been tested intensively in clinical settings. GLV-1 h68 (proprietary name GL-ONC1) carries three separate transgenic expression cassettes (encoding β-glucuronidase, β-galactosidase, as well as the Ruc-GFP marker gene) inserted into a vaccinia virus (VACV) backbone derived from the *Lister* strain which has demonstrated its safety throughout years serving as a major smallpox vaccine. These triple insertions reduce the replication of GLV-1 h68 in healthy cells and favor its replication in tumor cells [11, 12]; beyond they also allow the monitoring of virus activities in cancer patients [13]. As this oncolytic virus is not targeted to a specific type of tumor, oncolytic activity has already been detected in a broad spectrum of tumor entities in preclinical models as well as in several clinical trials [13–16]. Moreover, combinatorial approaches with chemotherapy, radiation or targeted therapies have displayed synergistic antitumor activities [17–21].

Currently, there are three active clinical studies (NCT02759588, NCT02714374, NCT01766739) which employ GLV-1 h68/GL-ONC1. Virus delivery pathways include intraperitoneal, intrapleural, and intravenous delivery. Notably, early virus clearance constitutes a problem, especially when GLV-1 h68 is applied systemically/intravenously. As complement inhibition seems to play a crucial role in virus depletion following intravenous application [22], a new strategy is the application of an anti-C5-antibody (eculizumab) prior to virotherapy [NCT02714374]. Another recent approach to prevent intravascular virus clearance is to administer virus loaded cells as a carrier system for viral particles [23, 24]. Reasonable options for NENs constitute intravenous administrations as well as direct virus injections into the hepatic artery in case of liver involvement (NCT02749331, [9];). Further, intratumoral virus administrations or surgically guided administrations into the resection beds can be considered.

In this work, we now additionally have studied the combination of GLV-1 h68 with molecular targeted therapy (MTT). The mTOR inhibitor everolimus is approved as a treatment for advanced lung, pancreatic and intestinal NETs. This situation would be suitable for virotherapy to enter the clinical development in NEN therapy. Another option for MTT is the multi-kinase inhibitor sunitinib, which is approved for pancreatic NETs. However, recent studies show significantly longer progression free survival with everolimus used as a first line MTT in pancreatic NETs compared to sunitinib. Also, everolimus MTT was found to be significantly more efficient in non-pancreatic NETs, which is why the combinatorial treatment of GLV-1 h68 with everolimus was investigated here in a preferred way [25–27].

In this study, tumor cell lines originating from pancreatic NETs, lung NETs and intestinal NECs were evaluated for their susceptibility to vaccinia virus-mediated virotherapy. For this purpose, the lytic activity of GLV-1 h68 was measured, viral gene expression was visualized and virus replication was quantified. Beyond that, also a combinatorial treatment regimen being set up for the conjoint usage of GLV-1 h68 and everolimus was studied for its ability to deplete NEN tumor cells; besides, possible interactions between everolimus and replication of the oncolytic virus GLV-1 h68 were investigated also.

## Methods

### Oncolytic virus

The oncolytic vaccinia virus GLV-h168 was kindly provided by Genelux Corporation (San Diego, CA, USA). GLV-1 h68 is a genetically engineered OV originating from the vaccinia *Lister* strain and also known under the proprietary name GL-ONC1 [11]. It was genetically modified by inserting three transgenes allowing therapeutic monitoring in its genome; RUC-GFP is employed for monitoring via fluorescence microscopy in this study.

### NET/NEC cell lines

The six cell lines derived from NENs are outlined in Table 1. H727, UMC-1, QGP-1, and NEC-DUE1 cells were maintained in RPMI-1640 medium (Gibco, Waltham, MA, USA) supplemented with 10% fetal calf serum (FCS, Biochrom, Berlin, Germany). BON-1 cells were cultured in Dulbecco’s modified Eagle’s Medium (DMEM, Sigma-Aldrich, St. Louis, MO, USA) supplemented with 10% FCS and HROC-57 cells required DMEM/F12 medium (Gibco) with 10% FCS. CV-1 African green monkey kidney cells were purchased from ATCC (CCL-70) and cultured in DMEM supplemented with 10% FCS. All cells were cultured at 37 °C and 5% CO_2_ in a humidified atmosphere and seeded in 6- and 24-well plates for the respective assays.

**Table 1:** NET/NEC cell lines employed in this study on GLV-1 h68 vaccinia virus therapy of neuroendocrine tumors

Cell line	Origin	Source	Reference
H727	Lung NET	ATCC	[28]
UMC-11	Lung NET	ATCC	[29]
BON-1	Pancreatic NET	Dr. Ulrich Renner, MPI Psychiatry, Munich, Germany	[30]
QGP-1	Pancreatic NET	JCRB (Japanese Collection of Research Bioresources Cell Bank)	[31]
HROC 57	Colon ascendens NEC	Dr. Michael Linnebacher, University Hospital Rostock, Germany	[32]
NEC-DUE1	Liver metastasis of a NEC at the gastroesophageal junction	Dr. Andreas Krieg, University Hospital Duesseldorf, Germany	[33]

### Virus infections and everolimus treatment

For infection, cells were seeded 24 h before. GLV-1 h68 was diluted in the respective amount of DMEM supplemented with 2% (v/v) FCS to prepare the infection medium. The dilution ratio was calculated to ensure infection with a specific multiplicity of infection (MOI, effector target ratio, i.e. viral particles per cell). Cells were rinsed with phosphate buffered saline (PBS, Sigma-Aldrich) prior to infection, shortly before the respective amount of infection medium was added. Virus infection was allowed to take place for 1 h with swaying every 15 min. Then, infection medium was replaced with normal cell culture medium. Mock treatment was conducted with DMEM supplemented with 2% (v/v) FCS. For sole treatment with everolimus (Sellekchem, Munich, Germany), cell culture medium was replaced with medium containing everolimus at the respective concentration at 24 h post cell seeding. For combinatorial treatment with GLV-1 h68 and everolimus, infection medium was replaced with cell culture medium containing everolimus in the respective concentration.

### Cell viability assays

To assess tumor cell viabilities at 72 and 96 h post infection (hpi), the Sulforhodamine B (SRB) assay was employed. This viability assay measures cell density compared to mock treatment by quantifying the number of adherent (viable) cells [34]. For this purpose, NET/NEC cells were seeded in 24-well plates and infected with OV, mock treated, treated with everolimus or OV and everolimus together. At the respective time point of analysis (at 72/96 hpi), cells were fixed with 10% (v/v) trichloroacetic acid (Carl Roth, Karlsruhe, Germany) after rinsing them with 4 °C cold PBS. Fixation was allowed for at least 30 min at 4 °C. Next, cell cultures were washed with water. Then, fixed cells were stained with SRB dye (0.4% (w/v) in 1% (v/v) acetic acid; Sigma-Aldrich) for at least 10 min and rinsed afterwards with 1% (v/v) acetic acid (VWR, Radnor, PA, USA) to remove unbound SRB dye. After drying for another 24 h, 10 mM TRIS base (pH 10.5; Carl Roth) was added to solve remaining SRB dye. To measure the amount of bound SRB dye, the absorbance of the inoculum at a wavelength of 550 nm was determined in duplicates (using a Tecan Genios Plus Microplate Reader). As the SRB dye binds to cellular proteins, the absorbance correlates with cell density. In the figures, cell density of mock treated cells was adjusted as 100%; percentages refer to mock treatment.

### Microscopy

For microscopy, an Olympus IX 50 microscope with a PhL phase contrast filter and a fluorescence filter for GFP detection was used. Pictures were taken with the F-View Soft Imaging System (Olympus) and were colored and overlaid afterwards with the analySIS image-processing software and Apple Preview 10.0 software.

### Real-time cell monitoring assay

H727 cells were seeded in 96-well plates (E-Plate 96, Roche Applied Science, Mannheim, Germany). The xCELLigence RTCA SP system (Roche Applied Science) was employed to observe impedance of the cell layer in 30 min intervals over 120 h. 24 h after seeding, cells were infected with GLV-1 h68 using MOIs 0.1 and 0.25 or treated with 0.1% (v/v) Triton for lysis control. The measured impedance was used to calculate Cell Index values with the RTCA Software (1.0.0.0805).

### Virus plaque assays

Plaque assays were conducted in order to determine the concentration of viral particles in cell cultures as described previously [11]. H727 and BON-1 cells were seeded in 6-well plates and infected with MOIs which led to approx. 50% reduction of tumor cell densities. One hour after virus infection, plates were carefully washed with PBS to remove all extracellular viral particles; then culture medium was added. Every 24 h and at 1 hpi, infected cells and medium were harvested by scraping them into the culture medium. Subsequently, the harvested samples were frozen at − 80 °C. For analysis of the samples, the CV-1 indicator cells were infected with the frozen samples. For this purpose, thawed samples were titrated in duplicates in 10-fold dilutions (10^− 1^ to 10^− 6^) on the indicator cells. Cells were incubated for 1 h and plates were moved every 15 min to ensure sufficient virus infection. Next, cells were overlaid with 1 ml of 1.5% (w/v) carboxymethylcellulose (CMC, Sigma-Aldrich) in DMEM with 5% (v/v) FCS and 1% (v/v) Pen/Strep per well. As the CMC medium prevents viral spread through the culture medium, each infective viral particle creates a plaque by radial infective spread after 48 h. After 48 h, cell layers were stained with crystal violet staining solution (0.1% (w/v) in 5% (v/v) ethanol, 10% (v/v) formaldehyde, Fluka Chemie AG) for 4 h. Then, the culture plate was washed with water and plaques could be counted. With the plaque count and titrated dilutions, viral titers (plaque forming units (PFU) per ml) could be calculated.

### Statistical analysis

Results of SRB viability assays regarding GLV-1 h68 monotherapy were found to be equally distributed with inhomogeneous variations and were statistically analyzed using a Welch’s ANOVA and Dunnett T3-test for inhomogeneous variations. For combinatorial therapy with everolimus, a two tailed t-test for independent samples with inhomogeneous variations was conducted for samples requiring statistical analysis. *P* values ≤0.05 were set statistically significant and IBM SPSS Statistics Version 26 was used.

## Results

### Virotherapy with GLV-1 h68

First, effects of a monotherapy of the six NET/NEC cell lines employing the vaccinia virus vector GLV-1 h68 were studied. In this purpose, SRB viability assays were conducted to evaluate cytostatic and cytotoxic effects of the OV on neuroendocrine cancer cells and to identify oncolysis-sensitive and -resistant tumor cell lines. Further, microscopic fluorescence pictures were taken to visualize oncolysis and directly detect and prove virotherapeutic vector-based transgene (GFP) expression. Next, a real-time cell monitoring assay was employed to distinguish between cytostatic and cytotoxic nature of the effect and study the dose dependency of this circumstance. Finally, the production of viral progeny, which forms the basis of the intratumoral infectious spread of an OV, was studied by assessing virus titers sequentially over time.

### Oncolysis with GLV-1 h68

**Fig. 1:**
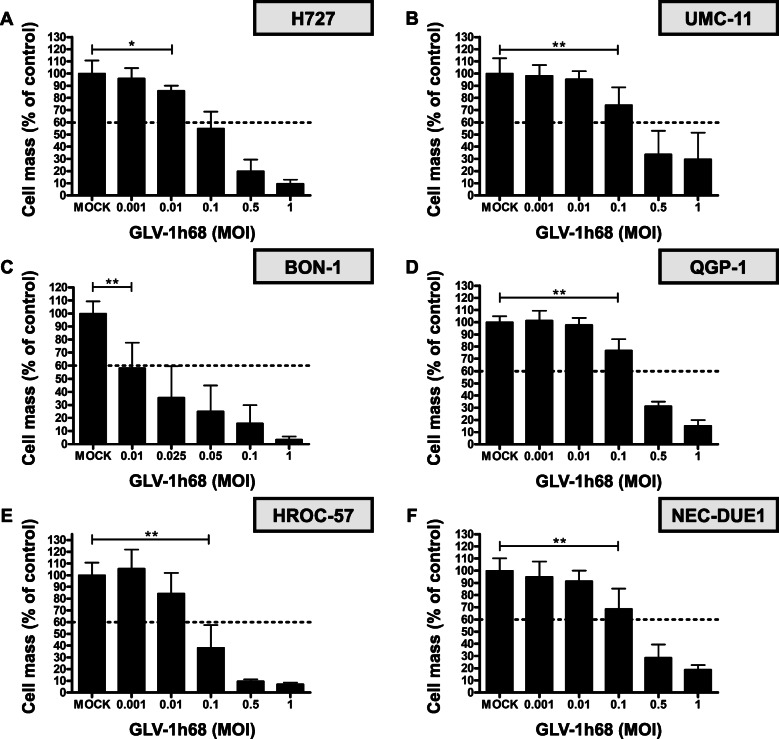
Oncolysis with GLV-1 h68. SRB viability assays employing oncolytic vaccinia virus vector GLV-1 h68 on the NET/NEC cell line panel of six different tumor cell lines originating from different neuroendocrine neoplasms. Lung NET cell lines are shown in the upper panel (**a**, **b**), pancreatic NET cell lines in the middle (**c**, **d**), and intestinal NEC cell lines in the lower panel (**e**, **f**). Analysis was performed at 96 hpi. H727, BON-1 and HROC-57 cells were found to be highly permissive; UMC-11, QGP-1, and NEC-DUE1 cells were classified as permissive. BON-1 cells exhibited a quite strong response, requiring only MOI 0.01 to reach the threshold of 60% remaining tumor cells. Four independent experiments (six for UMC-11 cells) were carried out in quadruplicates; bars show mean and SD. The lowest MOI being significantly superior to mock treatment is indicated with * *p* < 0.01 or ** *p* < 0.001. Higher MOIs of the same cell line were also found to be significantly superior to mock treatment

All NET/NEC cell lines were infected with multiplicities of infection (MOIs) of GLV-1 h68 in logarithmic steps, ranging from 0.0001 to 1. Taking the first results of the SRB viability assays into account, the MOIs were modified by adding MOI 0.5 instead of MOI 0.0001 for all cell lines except BON-1; MOIs 0.025 and 0.05 were added for BON-1 cells, while MOIs 0.0001 and 0.001 were left out. A threshold for clinically relevant anti-tumor activities was set at 60% of tumor cells being residual in SRB viability assays after an infection period of 96 h (Fig. 1, dotted horizontal lines). Three categories to classify cellular response to GLV-1 h68 virotherapy were introduced: (i) highly permissive cell lines, meeting the 60% threshold with MOI 0.1 or less after 96 h; (ii) permissive cell lines requiring MOI 0.5 to meet the threshold at 96 hpi, and (iii) resistant cell lines which required more than MOI 0.5 to meet the threshold at 96 hpi.

It was found that GLV-1 h68 is able to infect and kill all six NET/NEC cell lines, requiring different MOIs for the same effect. For all tumor cell lines, a dose dependency was observed, meaning that a higher MOI resulted in a lower number of residual tumor cells at the end of the observation period, i.e. at 96 hpi. As a result, three highly permissive, three permissive and no resistant cell lines could be identified. BON-1 pancreatic NET (pNET) cells were found to be most sensitive to GLV-1 h68-mediated oncolysis, exhibiting a remaining tumor cell mass of 60% at 96 hpi when using a MOI of only 0.01 (Fig. 1c). For all other NET/NEC cell lines higher MOIs had to be applied in order to meet the 60% threshold at 96 hpi: MOI 0.1 was sufficient for H727 and HROC-57 cells (Fig. 1a and e); accordingly, BON-1, H727, and HROC-57 cells were classified as highly permissive. In contrast, UMC-11, QGP-1, and NEC-DUE1 cells required MOI 0.5 and were classified as permissive. An equal response pattern could be found in all three permissive cell lines. All three showed a significant reduction of remnant tumor cells with MOI 0.1 and met the threshold with MOI 0.5. Finally, a remaining tumor cell count of approx. 15% was reached with MOI 1 in all three cell lines (Fig. 1b, d, and e).

Overall, GLV-1 h68 was able to reduce the tumor cell masses to a minimum of less than 10% in 3 out of 6 NET/NEC cell lines.

In summary, no neuroendocrine cancer cell line turned out to be resistant to GLV-1 h68-mediated oncolysis. The three highly permissive cell lines were found to be BON-1 originating from a pNET, HROC-57 originating from a colon NEC and the lung NET derived cell line H727. Given that the three other cell lines showed very similar responses, no obvious relation between anatomical origin and treatment response could be identified in this experiment.

### Microscopy of GLV-1 h68-mediated NET/NEC cell oncolysis

**Fig. 2:**
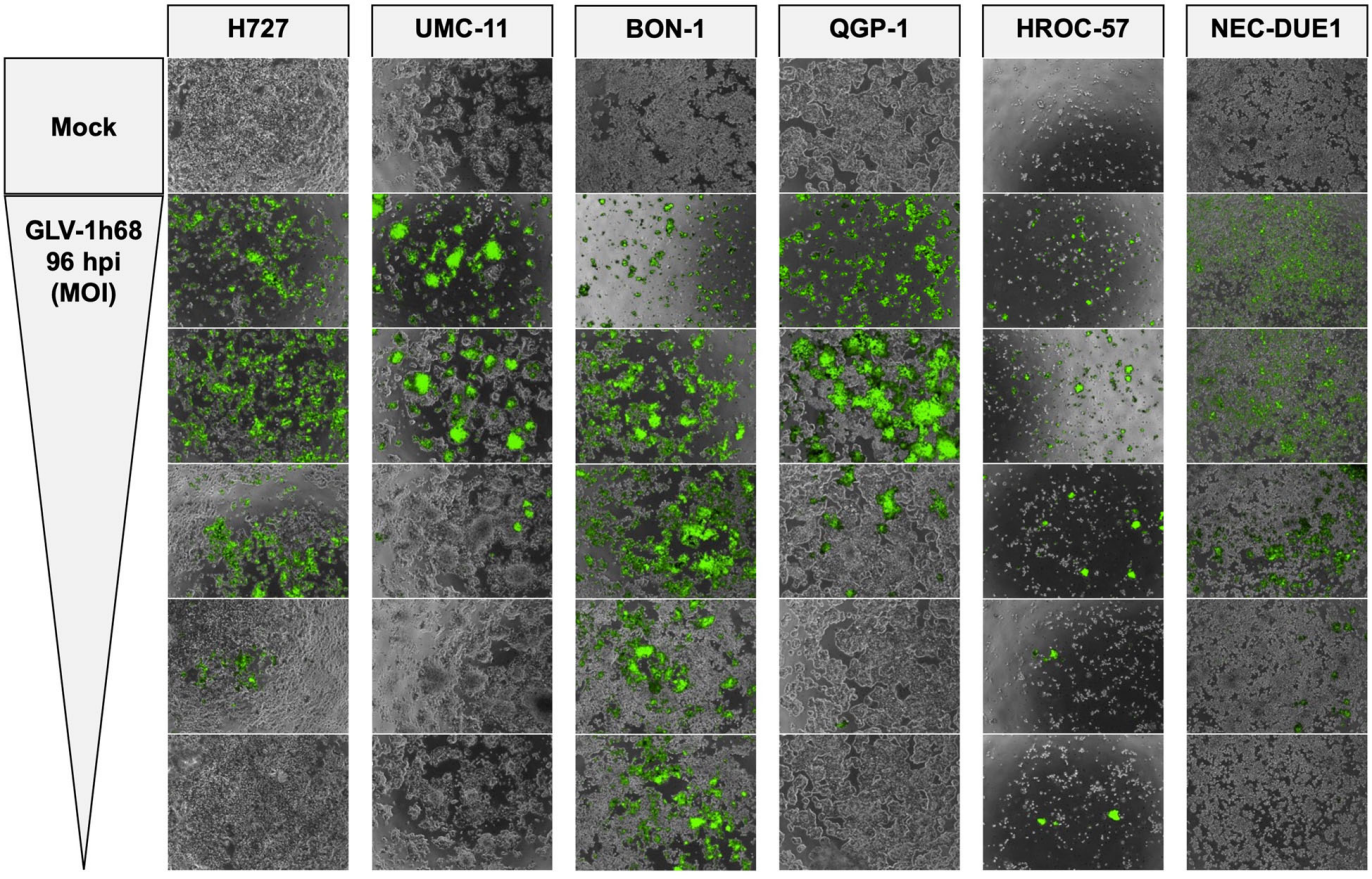
Microscopy of viral transgene expression. Fluorescence microscopy of the NET/NEC panel infected with oncolytic vaccinia virus vector GLV-1 h68. Phase contrast and fluorescence pictures were taken at 96 hpi and overlaid. From top to bottom, MOIs decrease and match the MOIs used in the respective SRB viability assays (Fig. 1). When using higher MOIs, infected cells displayed higher transgene expression. In BON-1, HROC-57, and QGP-1 cells, being highly permissive or permissive to GLV-1 h68 oncolysis, tumor cell killing already had been accomplished at 96 hpi resulting in lower GFP signals using high MOIs. No viral transgene expression could be observed in mock samples

As GLV-1 h68 encodes a fluorescent GFP transgene for therapeutic monitoring, microscopic pictures were taken to prove viral infection and replication via transgene expression and observation of cell layer densities (Figs. 2 and S1). The same MOIs as in the SRB viability assay (Fig. 1) were applied. As a result, a loss of cell density could be observed in all infected neuroendocrine cancer cell lines, consistent with results from the SRB viability assay, where all tumor cell lines were found to respond to virus infections. Moreover, all analyzed NET/NEC cell lines were found to express the GFP transgene when being infected with GLV-1 h68. Of note, lower cell confluency and intensities of the fluorescence signals were found to correlate to the MOIs being applied (Figs. 2 and S1). This does not apply for HROC-57 cells, as the confluency was also low in uninfected cells (mock). However, with the highly permissive cell lines (H727, BON-1, HROC-57), the highest MOI displayed lower transgene expression, most likely because of a high rate of oncolysis and therefore a lower cell count expressing the fluorescent GFP transgene. This phenomenon is also visible with permissive QGP-1 cells and on the respective pictures taken at 72 hpi, although to a lesser extent (Figure S1). Mock treatment did not display any fluorescence at all.

### Real-time cell monitoring

To precisely investigate the nature of the effect of GLV-1 h68 on neuroendocrine cancer cells, a real-time cell monitoring assay was employed. The lung NET cell line H727 was picked as representative cell line because it showed a stable, average response to GLV-1 h68 in the experiments described above. Two MOIs (0.1 and 0.25), which resulted in remaining tumor cell numbers of around 50% according to SRB viability assay performed at 96 hpi, were chosen for infection. The xCELLigence RTCA assay measures cellular impedance, which was shown to correlate with cell number, cell size/morphology and cell attachment quality [35]. Taking the previous SRB viability assays and applied cell lysis control with Triton X-100 into account, the Cell Index can be seen as a surrogate for cell viability in this context.

**Fig. 3:**
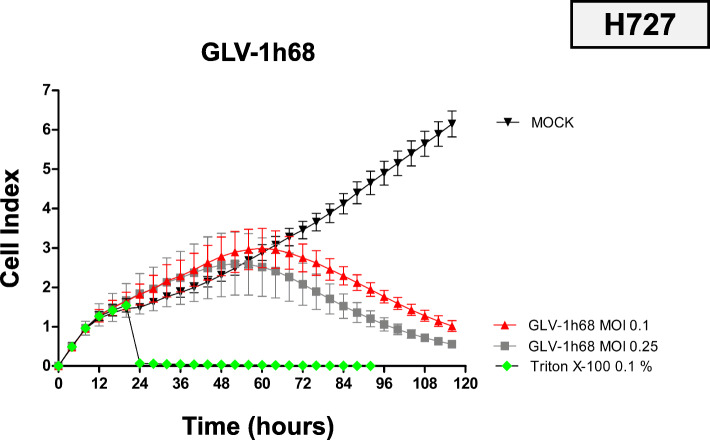
Real time cell monitoring of OV monotherapy and combinatorial approaches. Development of tumor cell viability during the treatment. Continuous measurement of cellular impedance (Cell Index) was conducted via xCELLigence assay over 120 h. Different treatments (GLV-1 h68 infection, Triton X-100, or mock treatment) were performed at 24 h and H727 lung NET cells were employed. Tumor cells were infected with different MOIs of GLV-1 h68. GLV-1 h68 was found to exhibit dose dependent cytotoxic effects on the NET cells, showing a reduction of cellular impedance over time which was found to be pronounced with higher MOI (0.25). The experiment was carried out in quadruplicates, bars show mean and SD

Different treatment modalities were initiated at 24 h after cell seeding. As expected, treatment with the cell lysis control Triton X-100 immediately resulted in a complete tumor cell lysis (Fig. 3; green dotted line). In contrast, virus infections showed similar results to mock treatment in the first 24 hpi. In the further course of the experiment, the impedance of infected cells decreased continuously, indicating not only a cytostatic but also a cytotoxic effect of GLV-1 h68. The higher MOI (0.25) results in lower cell viability in the end, but not in a faster mechanism of action, also showing the first impairment of tumor cell growth at 24 hpi and the peak of cell viability at 36 hpi (Fig. 3; line with grey squares). Taken together, GLV-1 h68 was proven to exhibit a pronounced oncolytic effect on the neuroendocrine tumor cell line H727 and also a dependency on the infectious dose being applied. Thus, findings of the SRB viability assay could be confirmed.

### Virus titer quantification

**Fig. 4:**
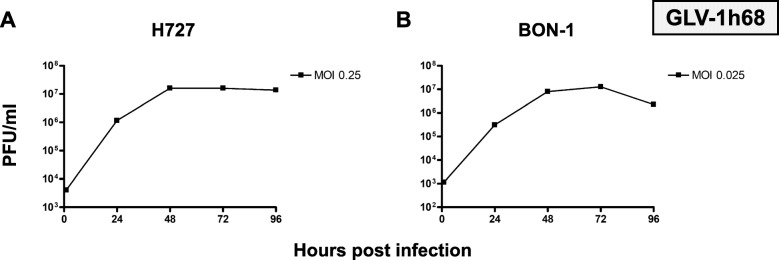
Virus quantification of OV monotherapy. Virus titer growth curves performed for oncolytic vaccinia virus vector GLV-1 h68 using representative NET cell lines of lung (H727) and pancreatic (BON-1) origin. For both cell lines, a 10,000-fold rise in viral titers could be observed during the first 48 h and titers higher than 10^7^ PFU/ml were reached. Then, viral growth was found to stagnate, being due to oncolytic reduction of virus host cell counts. Plaque forming units (PFU) were determined every 24 h; samples were analyzed in duplicates; experiments were performed twice; one representative result is shown

As the production of viral progeny is an important step in the underlying mechanism of oncolytic virotherapy, virus titers obtained by neuroendocrine cancer host cells were sequentially determined every 24 h during the whole period of infection. Hence, the lung NET cell line H727 (Fig. 4a) and the pNET cell line BON-1 (Fig. 4b), which was found to be the tumor cell line being most sensitive to GLV-1 h68 treatment, were picked to further investigate tumor cells being established from different anatomical origins. Both NET cell lines were infected with MOIs achieving around 50% reductions of tumor cell counts in the SRB viability assays. Shortly after virus infection, all extracellular viral particles were removed so that only viral particles which had already entered the cells after a 1-h infection period could produce viral progeny.

As a result, high levels of viral replication could be detected in both tumor cell lines (Fig. 4). Titers over 10^7^ plaque forming units (PFU)/ml were easily reached within 72 h. A stagnation of virus titer growth could be observed for H727 after 72 h and a reduction of viral titer between 72 and 96 h was detected with BON-1 cells.

### Combinatorial treatment with everolimus

Next, a combinatorial treatment with the mTOR inhibitor everolimus was evaluated by comparing a combinatorial approach (GLV-1 h68 + everolimus) to GLV-1 h68 monotherapy. In this purpose, SRB viability assay and virus quantification were conducted.

### Oncolysis with GLV-1 h68 and everolimus

SRB viability assays were carried out using the lung NET cell line H727 and the NEC cell line NEC-DUE1, which both are tumor cell lines being generated from different anatomical origins. H727 cells were classified as highly permissive to GLV-1 h68 monotherapy whereas NEC-DUE1 cells were classified as permissive (Fig. 1). Again, MOIs leading to around 50% tumor cell reductions were chosen (0.1 and 0.25 for H727; 0.25 and 0.5 for NEC-DUE1). Everolimus was administered in concentrations of 1 nM for H727 cells and 0.25 nM for NEC-DUE1 cells, respectively.

**Fig. 5:**
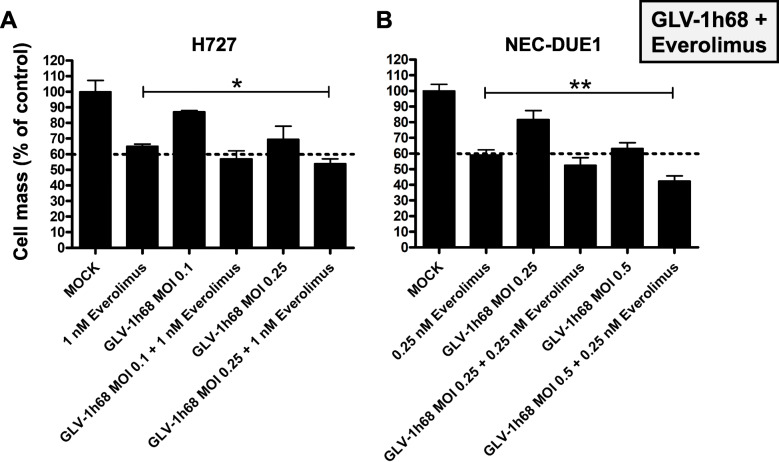
Cytotoxicity of combinatorial therapy. SRB viability assays employing the mTOR inhibitor everolimus, oncolytic vaccinia virus vector GLV-1 h68 and combination of both. H727 cells originating from a lung NET and the NEC-derived NEC-DUE1 cell line were employed and analysis was performed at 96 hpi. With both cell lines, combinatorial treatment with everolimus was found to be slightly more effective than single agent treatment with either everolimus or GLV-1 h68 alone. In both cell lines and for both MOIs tested, the addition of everolimus to GLV-1 h68 further reduced the remaining tumor cell count. Experiments were carried out in quadruplicates; bars show mean and SD. * *p* < 0.01; ** *p* < 0.001

As a result, the addition of GLV-1 h68 to sole everolimus treatment was found to be able to further reduce the remaining tumor cell count (Fig. 5). This was observed in both cell lines tested and with both MOIs employed in each cell line. With both cell lines, no statistical significance was found for the addition of the respective lower MOI to Everolimus treatment alone (*p* > 0.05). By adding MOI 0.25 for H727 cells and MOI 0.5 for NEC-DUE1 cells, the combinatorial treatment was able to reduce tumor cells significantly more than Everolimus alone (Fig. 5).

With H727 cells, the addition of MOI 0.25 to Everolimus reduced the remaining tumor cell count by 11% from 65 to 54%. Interestingly, the benefit of the combinatorial therapy appeared to be more pronounced in NEC-DUE1 cells. By adding MOI 0.5 to Everolimus treatment alone, the remnant tumor cells were reduced by 17% from 59 to 42%. However, the extent of this effect was limited, thereby not representing any additive mechanism of action.

### Virus titer quantification

**Fig. 6:**
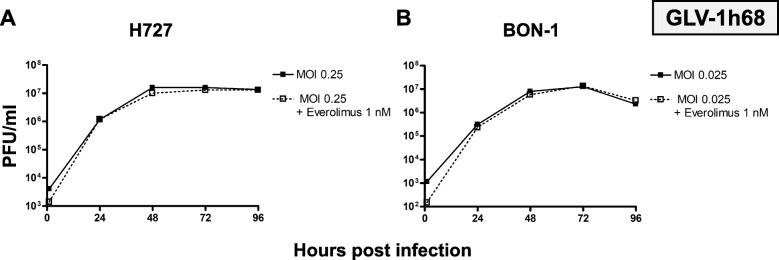
Virus quantification of the combinatorial approach. Virus titer growth curves were performed with H727 and BON-1 tumor cells under the same conditions in presence of everolimus (added at 1 hpi). Previous results from monotherapy (Fig. 4) are shown (solid lines). Interestingly, everolimus did not alter viral replication in any significant way (dotted lines). Plaque forming units (PFU) were determined every 24 h; samples were analyzed in duplicates; experiments were performed twice; one representative result is shown

To investigate whether everolimus has any impact on virus replication, virus titers were assessed when GLV-1 h68 was employed in a combinatorial setting with everolimus (Fig. 6, dotted lines). In both NET cell lines (H727, BON-1), where virus replication was determined previously, everolimus did not affect the production of viral progeny in any way.

Taking the results from both assays into account, the final benefit of the combinatorial therapy after 96 h is visible but only small. Everolimus did not limit virus replication in a particular way. Given that evidence base, the combinatorial therapy of GLV-1 h68 with everolimus was not found to be inferior to either monotherapy and can be regarded as a possible future combinatorial treatment option for metastatic neuroendocrine cancer.

## Discussion

Oncolytic virotherapy constitutes a novel therapeutic strategy to overcome treatment limitations and resistance in advanced stage tumors. Its mechanism of action comprises a tumor selective viral infection and subsequent oncolysis of tumor cells. Tumor selectivity of vaccinia viruses (VACVs) relies on multiple mechanisms which are closely related to the underlying characteristics of cancer. Most tumor cells fail to activate signaling pathways like interferon (IFN) or apoptosis pathways as a response to viral infection. Several other mechanism for tumor selectivity of VACVs have been described [36]. By selecting the most efficient virus strain and inserting several genes in different replication cassettes, GLV-1 h68 was modified to be attenuated in healthy cells and its replication was found to be mainly selective to tumor cells. In line with the basic characteristics of VACVs, GLV-1 h68 has the advantage of a stable cytoplasmic replication which avoids further virus-driven mutations in cancer cells or healthy cells [37]. In addition, the excellent safety profile of these VACVs is marked with years of clinical experience serving as smallpox vaccines as well as a preclinically well-established replication cycle [38]. Further, VACVs have no natural pathogenic potential in humans.

However, the key mechanism of oncolytic virotherapy is thought to be a secondary immune response induced by the inflamed lytic tumor microenvironment. The release of tumor antigens and inflammatory cytokines disables immune evasion mechanisms of the tumor and facilitates profound antitumor immune responses [39]. This effect was observed earlier when it was found that not only VACV-injected melanoma metastases decreased in size, but also non-injected distant lesions responded to virotherapy with a granulocyte-macrophage colony-stimulating factor (GM-CSF)-expressing vaccinia virus [40]. Both, a response of the innate immune system mediated by NK-cells, neutrophils and macrophages as well as an adaptive immunity facilitated by antigen-presenting cells and subsequent tumor-infiltrating CD8+ cells have been described after GLV-1 h68 treatment [41]. Obviously, this secondary immune-mediated mechanism is complicated to mimic in an in vitro setting. However, since GLV-1 h68 and other VACVs were reported to induce immunogenic cell death previously, the extent of direct tumor cell lysis can be regarded as a crucial factor in initiating an antitumor immunity [42, 43].

In this work, the potential of GLV-1 h68 to kill cells originating from neuroendocrine cancer has been demonstrated. GLV-1 h68 exhibited stable cytotoxicity throughout neuroendocrine cancer cells from several anatomical origins (Fig. 1). Susceptibility to GLV-1 h68 treatment was found to be dose dependent. Different responses of the variety of tumor cell lines was noted but could not be tracked back to a certain anatomical origin. In summary, three cell lines were found to be highly permissive, three were classified as permissive, and no cell line was found to be resistant to GLV-1 h68 monotherapy.

It was shown earlier that cellular response to GLV-1 h68 treatment depends on pleiotropic factors such as transcriptional patterns, cellular innate immunity pathways, efficiency of viral replication or proliferation rate [44]. Also, viral cytotoxicity was correlated with a strong transgene expression. Highly permissive cell lines (H727, BON-1, HROC-57) displayed GFP expression even at very low MOIs, whereas transgene expression was only observed with higher MOIs in permissive cell lines (UMC-11, QGP-1, NEC-DUE1) (Fig. 2). For the representative NET cell line H727, a fast mechanism of action of GLV-1 h68 therapy could be proven, resulting in a strong cytolytic response beginning as early as 36 h after virus infection (Fig. 3).

Moreover, a strong virus replication was shown in both NET cell lines tested, reaching virus titers higher than 10^7^ PFU/ml at 72 hpi (Fig. 4). The stagnation in virus titer growth after 72 h was explained by the efficient oncolytic depletion of tumor cells, resulting in significantly lower numbers of host cells being available for viral replication. Even a virus titer reduction from 72 to 96 h could be observed in BON-1 cells (Fig. 4b), since BON-1 cells were found to be most permissive to tumor cell killing. In summary, efficient production of viral progeny creates the basis for viral spread throughout the tumor, subsequent virus infection, following immunogenic cell death and induction of systemic anti-tumor immune responses.

Taken together, these results provide evidence for significant oncolytic effects in neuroendocrine cancer cells obtained by the vaccinia virus-based vector GLV-1 h68. Comparing these results to other OVs already tested in neuroendocrine neoplasms, GLV-1 h68 showed favorable cytotoxicity for pNETs and NECs. The oncolytic herpes simplex virus T-VEC, which is clinically approved for treatment of advanced melanoma, was found to be particular effective in lung and pancreatic NETs previously, thereby requiring lower MOIs than GLV-1 h68 for a relevant cytotoxicity [7]. Another OV which is currently under clinical investigation for treatment of liver metastases of NETs is the adenovirus AdVince (NCT02749331). In a previous preclinical evaluation, AdVince required a MOI of at least 1 to reduce cell viability of primary cells derived from metastatic small intestinal NETs [9]. The in vitro results for all three OVs are reasonably encouraging, however requiring further evaluation in animal trials or combinatorial treatment regimens.

This raises the question whether or not the combination with a clinically approved treatment, such as with the mTOR inhibitor compound everolimus, could augment effects of oncolysis in our panel of human NET/NEC cell lines, thus opening up novel treatment procedures for this unique tumor entity.

Everolimus was tested for its effect on viral replication to exclude any restrictions on replication of GLV-1 h68 in a combinatorial treatment regimen. It was found that everolimus does not influence GLV-1 h68 replication in a negative way (Fig. 4). However, combinatorial treatment was slightly superior and significantly more effective than any single agent treatment (Fig. 5). This makes this treatment modality feasible for further investigations. Of note, previous studies regarding the combinatorial therapy of VACVs with the mTOR inhibitor rapamycin, had resulted in the detection of synergistic effect. Both, everolimus and rapamycin target and inhibit mTORC1. The synergistic effects were explained by the effect of mTORC1 inhibition on antiviral immunity. It was found that mTORC1 downstream signaling via p70S6K/4E-BP1 influences cellular type I IFN response. Therefore, mTORC1 inhibition can make tumor cells more susceptible to VACV infection. In vivo, antiviral T-cell responses can be reduced by mTOR inhibitors, which also makes viral infections more effective [45–47]. These studies were conducted with malignant glioma models. In our study, these results could not be translated to neuroendocrine neoplasms, where the mTOR pathway might play another role in tumorigenesis. As both agents interfere with the immune system, further in vivo studies with immunocompetent animals have to be conducted to cover the whole range of mechanisms of action for this distinct combinatorial therapy.

Another possibility for combinatorial treatment with GLV-1 h68 could be the usage of the multi-kinase inhibitor sunitinib, which was shown to exhibit synergistic effects together with VACV virotherapy recently. This is explained by multiple mechanisms such as suppression of viral resistance, increased leakiness of tumor vasculature and therefore more effective viral infection and increased CD8^+^ T-cell recruitment [48].

For advanced NECs, the first line therapy constitutes a traditional chemotherapy employing cisplatin and etoposide. A previous study on pancreatic carcinoma showed benefits of the combination of GLV-1 h68 and cisplatin in nude mice [49]. In a phase 1 clinical trial, GL-ONC1, the proprietary name of GMP-derived material of oncolytic vaccinia virus GLV-1 h68, was added to cisplatin chemotherapy for head and neck squamous cell carcinoma, but only with limited success [14].

Nonetheless, the combination of immunotherapy and in particular virotherapy with cytotoxic chemotherapy is heavily discussed [50]. Advantages of this combination were found to be highly depending on the dosing scheme and time interval [51, 52]. It is also conceivable that cytotoxic chemotherapy limits the secondary antitumor immune response after virotherapy in immunocompetent animals and humans. In this line, ChemoVirotherapy combinations have not been examined in this work, but should be investigated in future work.

## Conclusions

In summary, this study created a very first basis for the development of GLV-1 h68 virotherapy in advanced neuroendocrine neoplasms. Future research has to confirm these preliminary findings in animal models and more realistic human tumor models, such as human NEN-derived organoids.

## Supplementary information

**Additional file 1 **: **Supplementary Figure S1**: Microscopy of viral transgene expression at 72 hpi. Representative phase contrast, fluorescence and overlay pictures of the NET/NEC panel infected with GLV-1 h68 taken at 72 hpi. When comparing with the pictures taken at 96 hpi (Fig. 2), GFP expression was found to be lower in all tumor cell lines at this earlier time point.

## Data Availability

All datasets generated and/or analysed during this study are available from the corresponding author on reasonable request.

## Abbreviations

ATCC: American Type Culture Collection
CD: Cluster of differentiation
CMC: Carboxymethylcellulose
DMEM: Dulbecco’s modified eagle’s medium
FCS: Fetal calf serum
GFP: Green fluorescent protein
GM-CSF: Granulocyte-macrophage colony-stimulating factor
hpi: Hours post infection
IFN: Interferon
MOI: Multiplicity of infection
mTOR: Mechanistic target of rapamycin
mTORC1: Mechanistic target of rapamycin complex 1
MTT: Molecular targeted therapy
NEC: Neuroendocrine carcinoma
NEN: Neuroendocrine neoplasia
NET: Neuroendocrine tumor
NK-cell: Natural killer cell
OV: Oncolytic virus
PBS: Phosphate buffered saline
Pen/Strep: Penicillin-Streptomycin
PFU: Plaque forming units
pNET: Pancreatic neuroendocrine tumor
RPMI: Cell culture medium developed at the Roswell Park Memorial Institute
RUC-GFP: Renilla luciferase-Aequorea green fluorescent protein
SD: Standard deviation
SRB: Sulforhodamine B
TRIS: Tris (hydroxymehtyl) aminomethane
VACV: Vaccinia virus
