# Supplementary figures and images for: Oncolytic vaccinia virus GLV-1h68 exhibits profound antitumoral activities in cell lines originating from neuroendocrine neoplasms
Source: BMC Cancer. 2020 Jul 6;20:628. doi: 10.1186/s12885-020-07121-8 (PMC7339398; doi:10.1186/s12885-020-07121-8)

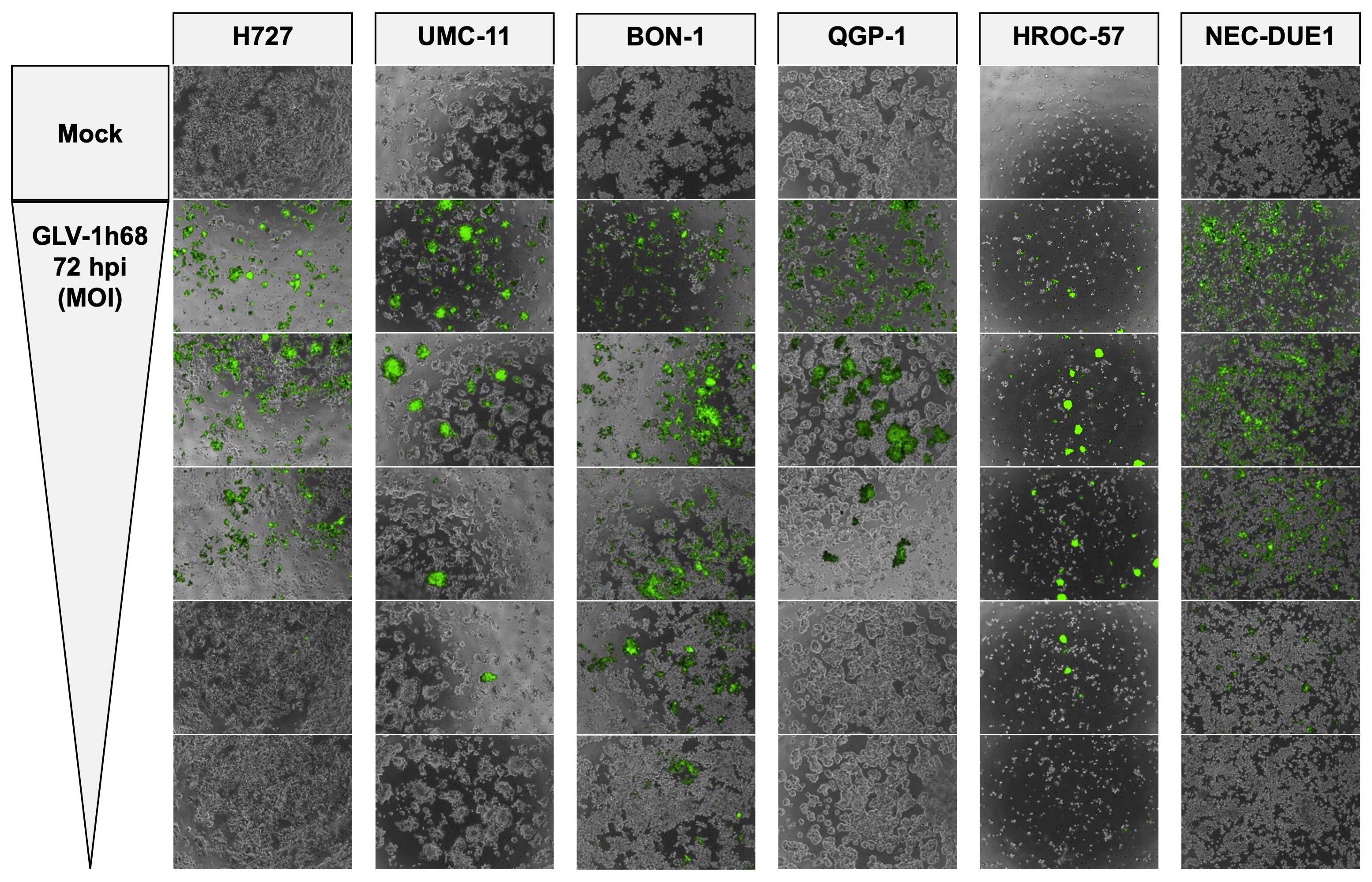

Supplement: Supplementary file 1 — Additional file 1 : Supplementary Figure S1: Microscopy of viral transgene expression at 72 hpi. Representative phase contrast, fluorescence and overlay pictures of the NET/NEC panel infected with GLV-1 h68 taken at 72 hpi. When comparing with the pictures taken at 96 hpi (Fig. 2), GFP expression was found to be lower in all tumor cell lines at this earlier time point. [file 12885_2020_7121_MOESM1_ESM.jpeg]
